# The Impact of *Chlorella vulgaris* Fortification on the Nutritional Composition and Quality Characteristics of Beef Burgers

**DOI:** 10.3390/foods13121945

**Published:** 2024-06-20

**Authors:** Basma R. Abdel-Moatamed, Alla-Eldeen M. A. El-Fakhrany, Nady A. A. Elneairy, Mohamed Mahmoud Shaban, Mohamed H. H. Roby

**Affiliations:** Food Science and Technology Department, Faculty of Agriculture, Fayoum University, Fayoum 63514, Egypt; bra11@fayoum.edu.eg (B.R.A.-M.); ama19@fayoum.edu.eg (A.-E.M.A.E.-F.); nan00@fayoum.edu.eg (N.A.A.E.); mmm12@fayoum.edu.eg (M.M.S.)

**Keywords:** algae, natural extracts, meat products, antimicrobial, antioxidant activity, bioactive compounds

## Abstract

*Chlorella vulgaris* (C.V) is known for its high protein and nutrient contents and has been touted as a potential functional ingredient in food products. For this study, beef burgers were formulated with varying levels of *Chlorella vulgaris* fortification (0%, 0.5%, 1%, and 1.5% by weight). The nutritional composition, including proximate analysis and mineral content, was determined for each treatment group. The quality characteristics evaluated included thiobarbituric acid (TBA), total volatile base nitrogen (TVBN), pH, and total acidity. The study included extracting the active substances from *Chlorella vulgaris* using three solvents, 50% ethanol, 95% ethanol, and water, to evaluate the effect on the antimicrobial and antioxidant activity. The results showed that the water extract had the highest total phenolic content (183.5 mg gallic acid equivalent per gram) and the highest flavonoid content (54 mg quercetin per gram). The aqueous extract had the highest content of total antioxidants, followed by the 95% ethanol and 50% ethanol extracts. Meanwhile, the 50% ethanol extract showed the best antimicrobial activity, while the aqueous extract had less of an effect on Gram-positive bacteria and no effect on *E. coli*. For the burger treatments, at the end of the storage period, it was observed that the microbial load of the treatments decreased compared to the control, and there was a high stability in the total volatile base nitrogen (TVBN) values for the treatments compared to the control, reaching a value of 22.4 at month 5, which is well above the acceptable limit, indicating spoilage. The pH values were higher for all of the treatments, with a lower total acidity for all of the treatments compared to the control. In conclusion, utilizing *Chlorella vulgaris* algae as a natural preservative to extend the freshness of burgers is a sustainable and innovative approach to food preservation. By harnessing the power of this green superfood, we not only enhance the shelf life of our food products but also contribute to a healthier and more environmentally friendly food industry.

## 1. Introduction

Food spoilage remains a significant challenge in the food industry, leading to substantial economic losses and food waste [1]. According to the world food program, one-third of food produced for human consumption is lost or wasted globally. This amounts to about 1.3 billion tons per year, worth approximately USD 1 trillion. In 2022, the food waste occurring at the retail, food service, and household levels was estimated at 19 percent of all food available to consumers [2]. Meat products, including burgers, were particularly susceptible to spoilage due to their high moisture content and perishable nature. This not only leads to economic losses but also raises environmental concerns [3]. Microbial growth and lipid oxidation are the primary causes of burger spoilage, leading to discoloration, off-flavor, and textural changes, ultimately rendering the product unsafe for consumption [4].

The beef burger is consumed as a fast food item and enjoyed globally [5]. Researchers have explored methods to enhance the nutritional value of meat burgers by fortifying them with essential vitamins and minerals like iron and zinc, which are naturally present in beef [6]. Additionally, incorporating functional ingredients such as antioxidants and probiotics can offer added health benefits to consumers [7]. A large and growing body of evidence supports that the intake of certain types of nutrients, specific food groups, or overarching dietary patterns positively influences health and promotes the prevention of common non-communicable diseases (NCDs). Healthy lifestyle and diet are associated with a significant reduction in the risk of obesity, type 2 diabetes, and cardiovascular diseases. Oxidative stress and the imbalance between pro-oxidants and antioxidants are linked to cardiovascular and metabolic diseases. Changes in the antioxidant capacity of the body may lead to oxidative stress and vascular dysfunction. Diet is an important source of antioxidants, while exercise offers many health benefits as well [8,9].

The food industry is constantly seeking innovative ways to increase the shelf life of products [10]. One such method that has been gaining attention is the use of *Chlorella vulgaris* algae in food preservation [11]. This green microalga is not only a powerhouse of nutrients but also possesses unique properties that can help prolong the freshness of perishable foods such as burgers [12]. By harnessing the natural antimicrobial and antioxidant properties of *Chlorella vulgaris* [13], food manufacturers can reduce the need for synthetic preservatives and additives, making their products more eco-friendly and appealing to health-conscious consumers [14]. The most pressing concerns in modern minced-meat processing are found in the research and development of natural compounds with strong antioxidant and antibacterial properties, as well as their Generally Recognized As Safe (GRAS) status [15,16]. In this case, *Chlorella vulgaris* may be an effective alternative.

*Chlorella vulgaris* is a unicellular green alga rich in bioactive compounds, including chlorophyll, carotenoids, and phenolic compounds. These compounds have been shown to possess antimicrobial activity against a wide range of foodborne pathogens [17,18,19,20]. In addition, *Chlorella vulgaris* is a good source of natural antioxidants, which can help scavenge free radicals and delay lipid oxidation in meat products [21]. In addition, they are microalgae rich in essential nutrients, including protein (50–60% as DW), vitamins (such as B vitamins and vitamin C), minerals (such as iron and calcium), and omega-3 fatty acids [22]. Its balanced amino acid composition and high protein content make it a valuable nutritional supplement. It has been traditionally used in East Asia as an alternative medicine for various health conditions [23], and it is considered safe for human consumption by the Food and Drug Administration (FDA) and GRAS certification [24].

Previous studies have demonstrated the effectiveness of *Chlorella vulgaris* in inhibiting the growth of various foodborne pathogens and spoilage microorganisms in different food matrices. For instance, research by [25,26] showed the antimicrobial potential of *Chlorella vulgaris* extracts against *Staphylococcus aureus* and *E. coli*. Additionally, the study by [26] highlighted the antioxidant capacity of *Chlorella vulgaris* in food systems.

The objective of this study is to evaluate the impact of incorporating different concentrations of Chlorella vulgaris powder (0.5%, 1.0%, and 1.5%) on the quality characteristics and nutritional value of beef burgers during frozen storage. By conducting a literature review and potential experimental research, this study aims to assess the shelf-life extension and enhanced nutritional profile of beef burgers with the addition of *Chlorella vulgaris.*

## 2. Materials and Methods

### 2.1. Raw Material

*Chlorella vulgaris* was obtained from the Algal Biotechnology Unit at Egypt’s National Research Centre in Giza, Egypt. The algae were grown indoors with the BG-11 medium of growth, according to [17]. Beef meat, salt, spices, onion, and soy protein were purchased from the local market in Fayoum, Egypt.

### 2.2. Chemicals, Solvents, and Reagents

Aluminum chloride (AlCl_3_), sodium carbonate, ethanol, hexane, hydrochloric acid, boric acid, glacial acetic acid, thiobarbituric acid reagent, and sulfuric acid were sourced from El-Gamhoreya Company (Cairo, Egypt). Methanol was purchased from Aldrich Co. (London, UK). Folin–Ciocalteu reagents were obtained from Sigma Chemical Co. (St. Louis, MI, USA). All of the other solvents and chemicals used in the study were of analytical grade.

In vitro diagnostic discs (Pasteur LBA, Giza, Egypt) of penicillin were used to test the susceptibility of the used microorganisms.

### 2.3. Media

The BG-11 medium was prepared according to [27]. LB broth and LB agar, total count agar, baird parker agar, potato dextrose agar, and MacConkey agar were prepared according to the methods described in [28].

### 2.4. Microorganisms

As indicator microorganisms for antimicrobial activity, two Gram-positive (*Bacillus subtilis* and *Staphylococcus aureus* ATCC 13565) and one Gram-negative bacteria (*Escherichia coli*, O:157 ATCC 1659) were utilized. All of the strains listed here were obtained as actively growing cultures from the Microbiological Resources Centre Cairo (MIRCEN), Faculty of Agriculture, Ain Shams University, Cairo, Egypt.

### 2.5. Methods

#### 2.5.1. Cultivation of *Chlorella vulgaris*

*C. vulgaris* was cultivated for 3 weeks at 25 °C under constant light in the BG-11 nutrition media. Microalgae cells were collected by centrifugation at 3000 rpm for 5 mins. The harvested biomass was dried in a hot-air oven at 55 °C and ground into fine powder. *C. vulgaris* was utilized to make crude extracts with various solvents [29].

#### 2.5.2. Determination of Chlorophylls and Carotenoids

Pigments were determined according to [30] using 96% methanol and recording the absorbance at 666, 653, and 470 nm, and the concentrations of the pigments were calculated by the following equations:

Chlorophyll a (mg/g)=(15.65 × A666)−(7.340×A653)

Chlorophyll b (mg/g)=(27.05 × A653)−(11.2×A666)

Total carotenoids (mg/g)=(1000 × A470)−[(2.860× Ca)−(129×Cb)]/245

where A666, A653, and A470 nm are the absorbances at the indicated wavelengths.

#### 2.5.3. Preparation of the *C. vulgaris* Extracts

The extracts were prepared using ethanol 95%, ethanol 50%, and water. In a conical flask, 10 g of *C. vulgaris* powder was mixed with 100 mL of each solvent, then placed at 25 °C for two days in a shaking water bath. The mixtures were then centrifuged at 10,000 rpm to filter them.

Re-maceration was performed three times over 48 h until the color of the mixture faded. The resulting filtrate was extensively dried in a hot-air oven at 40 °C for 30 min.

The extraction yield was calculated using the equation shown below:

$$\mathrm{Yield}\;(\%)=\frac{W1}{W2}\times 100$$

where *W*1 is the extract weight after the evaporation of the solvent and *W*2 is the *C. vulgaris* dry weight.

#### 2.5.4. Determination of the Total Flavonoid and Phenolic Contents

The total phenols in the crude extracts were determined using the Folin–Ciocalteu reagent as described by [31] and the results were represented as mg gallic acid/g of sample (GAE/g). The spectrophotometric approach described by [32] was used to assess the flavonoid content of the Chlorella preparations and was calculated as quercetin (mg/g dry weight).

#### 2.5.5. Antioxidant Activity of the *Chlorella vulgaris* Extract by Phosphomolybdate Assay

The method proposed by [33] was used to determine the total antioxidant capacity (TAC). In brief, a test tube containing 300 µL of *Chlorella vulgaris* extract (100 mg/mL) or normal ascorbic acid (200–800 µg/mL) was combined with 3 mL of phosphomolybdate. For 90 min, the test tube was covered and incubated at 95 °C. After allowing the mixture to cool to room temperature, the absorbance at 695 nm was measured. The blank was run in the same manner as the sample, but with methanol in place of the sample. The TAC was expressed in milligrams of ascorbic acid equivalent (AAE) per gram of extract.

#### 2.5.6. Antibacterial Assay

The antibacterial activity of *C. vulgaris* extracts was determined using the well diffusion method [34]. A 7 mm well was created inside the plate’s edge, and a 25–50 µL aliquot of *C. vulgaris* extract was placed into it. In the control treatment, instead of extract, the well was filled with 10% DMSO (dimethyl sulfoxide). *B. subtilis*, *S. aureus*, and *E. coli* were cultured on plates at 35 °C for 24 h and 37 °C for 48 h, respectively. Clear zones of inhibition (in mm) were measured.

#### 2.5.7. HPLC Conditions

HPLC analysis was carried out using an Agilent 1260 series. The separation was carried out using an Eclipse C18 column (4.6 mm × 250 mm i.d., 5 μm). The mobile phase consisted of water (A) and 0.05% trifluoroacetic acid in acetonitrile (B) at a flow rate of 0.9 mL/min. The mobile phase was programmed consecutively in a linear gradient as follows: 0 min (82% A); 0–5 min (80% A); 5–8 min (60% A); 8–12 min (60% A); 12–15 min (82% A); 15–16 min (82% A); 16–20 (82%A). The multi-wavelength detector was monitored at 280 nm. The injection volume was 5 μL for each of the sample solutions. The column temperature was maintained at 40 °C [35].

#### 2.5.8. Burger Samples Preparation

The constituents of the burger (beef meat, 65%; salt, 1.5%; ice water, 10%; spices, 0.5%; onion, 2%; fat, 10%; soy protein, 10%) were mixed, and then mixed with the dried *Chlorella vulgaris* at different levels (0, 0.5, 1.0, and 1.5%). Each treatment was mixed for 5 min using a homogeneous mixture. This mixture was shaped using a burger maker into disc pieces of 60 g and a thickness of 1 cm to obtain the burgers. Plastic packaging film was used to help maintain the shape of the burgers prior to freezing and storage at −20 °C.

#### 2.5.9. Chemical Composition of *Chlorella vulgaris* Powder and Burger Samples

The moisture content of the burgers was determined by drying ten grams of the sample at 100–105 for 6 h until the constant weight was as described by the AOAC [36]. The ash content was measured by incinerating 0.3 g of the burger sample in a muffle furnace at 550–600 °C until all of the organic matter was burned off, leaving the inorganic minerals. The residue was weighed and expressed as a percentage of the original sample weight.

The ether extract, representing the lipid content, was determined using a Soxhlet extraction apparatus. The dried burger sample was extracted with petroleum ether at 40–60 °C for 4 h. The solvent was then evaporated, and the remaining fat was weighed and expressed as a percentage of the original sample weight.

The protein content was determined by the Kjeldahl method, which measures the nitrogen content of the sample. The nitrogen was converted to protein using a conversion factor (typically 6.25 for meat products). The protein content was then expressed as a percentage of the original sample weight.

The mineral content for the algae was determined by analyzing the ash obtained from the ash content determination. Specific minerals, such as calcium, potassium, magnesium, and iron, were quantified using inductively coupled plasma (ICP).

The crude fiber content of each sample was determined by digesting the sample in a 1.25% H_2_SO_4_ solution, followed by a 1.25% NaOH solution; then, the remaining residue was dried, weighed, incinerated, and weighed again. The difference in weight before and after incineration represented the crude fiber content, expressed as a percentage of the original sample weight.

#### 2.5.10. Chemical Quality Attributes

Total volatile nitrogen (TVB-N) is a measure of the nitrogenous compounds that are volatile at a specific temperature, primarily used as an indicator of spoilage in meat and seafood products. The TVB-N was determined by distillation methods, where the volatile nitrogenous compounds were distilled off and absorbed in a boric acid solution. The solution was then titrated with a standard acid to determine the nitrogen content, expressed as mg of nitrogen per 100 g of sample, as described in [36].

The TBA value is used to assess the extent of lipid oxidation in food products, particularly meat and fish. It measures malondialdehyde (MDA), a secondary oxidation product. The sample was homogenized with a TBA reagent and incubated, forming a pink chromogen, which was measured spectrophotometrically at 532 nm. The TBA value was expressed as mg of MDA per kg of sample, indicating the level of oxidative rancidity.

For the pH determination, a homogenized sample of the burger was prepared, and the pH was measured using a calibrated pH meter. The pH value is crucial for understanding the quality, shelf life, and microbial stability of the product.

For the total acidity in the burgers, the sample was homogenized and titrated with sodium hydroxide 0.05 N using phenolphthalein as an indicator. The amount of sodium hydroxide used to reach the endpoint was recorded, and the total acidity was calculated and expressed as a percentage of the original sample weight, often as the lactic acid equivalent in the meat products.

#### 2.5.11. Microbiological Evaluation

##### Preparation of Samples for Microbiological Examinations

Samples were firstly cauterized by using a hot spatula, then the cauterized parts were removed by using sterilized scalpel and forceps. Under aseptic conditions, 10 g of each sample were aseptically transferred into a sterile blender flask containing 90 mL of 1% sterile peptone water and homogenized at 14,000 rpm for 2.5 min. The mixture was left for 15 min at room temperature in order to achieve homogenization. The contents of the flask were thoroughly mixed by shaking, and 1 mL was transferred into a separate sterile tube containing 1 g of 0.1% sterile peptone water, from which ten-fold serial dilutions up to 10^−7^ were prepared [37].

##### Determination of Aerobic Plate Count

An amount of 1 mL of the previously prepared dilution was aseptically transferred into a sterile Petri dish, then about 10 mL of standard plate count agar, previously melted and tempered at 45 °C, was added and thoroughly mixed in a horizontal position. After solidification, inoculated as well as control plates were incubated in an inverted position at 37 °C for 48 h. Colonies that grew were counted and expressed as colony-forming units per gram (CFUs/g) of the sample.

##### Determination of *Escherichia coli* (*E. coli*) Counts

From each of the previously prepared sterile dilutions, 1 mL aliquots were delivered into duplicate sets of Petri dishes, previously inoculated with 10 mL of sterile MacConkey agar. After the solidification, inoculated as well as control plates were incubated in an inverted position at 37 °C for 48 h. *E. coli* colonies were identified based on their characteristic morphology and confirmed by biochemical tests. Results were expressed as CFUs/g of the sample, reflecting the potential contamination and sanitation effectiveness.

##### Determination of Yeast and Mold Count

The yeast and mold count measures the presence of these fungi, which can spoil food and affect the quality. Previously prepared dilutions in 1 mL aliquots were delivered into duplicate sets of Petri dishes previously inoculated with 10 mL of sterile potato dextrose agar medium. After the solidification, inoculated as well as control plates were incubated in an inverted position at 25 °C for 72 h. Yeast and mold colonies were counted and reported as CFUs/g of the sample. This count helped assess the product’s shelf life and potential spoilage issues.

##### Determination of Staphylococcus Aureus Count

The diluted dilution was plated onto Baird-parker agar. Plates were incubated at 35 °C for 24–48 h. Duplicate plates were prepared for each dilution. *Staphylococcus* colonies, often identified by their black or gray appearance with clear zones, were counted and confirmed with coagulase tests. Results were expressed as CFUs/g of the sample, indicating the risk of staphylococcal food poisoning.

### 2.6. Statistical Analyses

The experiment was designed as a randomized complete block design with nineteen treatments and ten replicates [38]. The data analysis was performed using an analysis of variance following the general linear model procedure by using the Info Stat computer software package (version, 2012). Means were separated using Duncan’s multiple-range test at the 95% level of probability.

## 3. Results and Discussion

### 3.1. Chemical Composition of Chlorella vulgaris (% Dry Weight)

The moisture content, protein content, ether extract, and ash were determined, and the obtained results are presented in Table 1.

*Chlorella vulgaris* contained 10.00% moisture, 38.17% crude protein,13.00% ash, 8.08% crude ether extract, 9.35% crude fiber, and 21.40% carbohydrates. These results were consistent with the results published by [39,40], who reported on the chemical composition of *Chlorella vulgaris*.

Minerals are inorganic compounds required by the human body to function effectively and maintain excellent health. The macro- and micro-elements of *Chlorella vulgaris* on dry weight bases are presented in Table 1. As shown in Table 1, *Chlorella vulgaris* had a high content of Na, P, Mg, K, Fe, and Zn, which was correlated with the higher amount of ash. These findings were consistent with the data provided earlier by other scientists [41]. *C. vulgaris* is an excellent source of critical micronutrients because of its diverse mineral composition.

*Chlorella vulgaris* contains a high concentration of chlorophylls and carotenoids [42]. Chlorophyll is a naturally occurring green pigment that plants and algae need for photosynthesis. As shown in Table 1, the concentration of total chlorophyll in *C. vulgaris* was 38.16 mg/g dry weight, which agrees with [43]. Chlorophyll is one of the possible Chlorella antioxidants. Some research has demonstrated that chlorophyll and its derivatives have diverse therapeutic benefits, such as wound healing and as an anti-inflammatory agent [44].

Algae, plants, fungi, and bacteria create carotenoid pigments, which range in hue from yellow to red. Table 1 shows that the total carotenoids in *C. vulgaris* was 17.56 mg/g. Carotenoids’ health-promoting qualities are typically linked to their antioxidant activity [45].

### 3.2. Total Phenols and Flavonoids in Chlorella vulgaris Extracts

**Figure 1:**
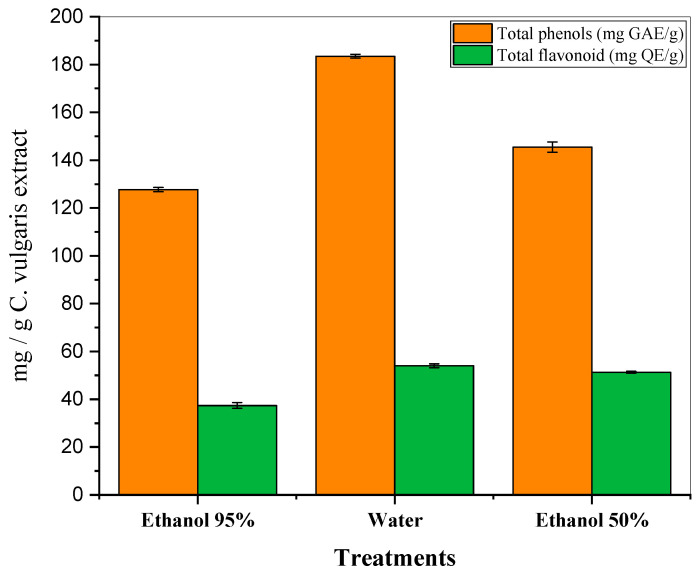
Total phenols and total flavonoids in *chlorella vulgaris* extracts.

The total phenols and flavonoids in the *Chlorella vulgaris* extracts were quantified by the Folin–Ciocalteu and AlCl_3_ methods, respectively. The results in Table 2 and Figure 1 present the yield extracts, total phenols, and total flavonoids in *Chlorella vulgaris* while extracted using the following three different solvents: 95% ethanol, water, and 50% ethanol.

From the results recorded in Table 2 and Figure 1, it is clear that the phenol content of the water extract was 183.5 mg GAE/g, while that of the 95% ethanol extract was 127.7 mg GAE/g and that of the 50% ethanol extract was 145.5 mg GAE/g. The water extract contained the highest concentration of phenols. The results of the determination of flavonoids using the AlCl_3_ technique showed that the content of flavonoids in the extracts of *C. vulgaris* was 54, 37.4 and 51.3 mg quercetin/g for the water, 95% ethanol, and 50% ethanol extracts, respectively. The water extract showed the highest amount of flavonoids.

The data strongly show that water is the best solvent for a higher yield of total phenols and total flavonoids extracted from *Chlorella vulgaris*. The lower efficacy of 95% ethanol in the extraction of phenols and flavonoids may be due to its lower polarity compared to the water and 50% ethanol, which are highly polar and are reputed for the better extraction of polar compounds such as phenolics and flavonoids. Therefore, solvent selection significantly influences the yield and content of bioactive compounds; in this study, water outperformed the 95% ethanol and 50% ethanol extracts.

Phenolic compounds have unique physical, chemical, and biological properties that make them effective as pharmaceuticals [46,47,48]. Phenols are also responsible for anticancer, antimicrobial, anti-inflammatory, and antiviral effects [49]. Furthermore, phenolic chemicals work as antioxidants by chelating ions of metals, limiting radical production, and enhancing the body’s natural antioxidant systems. Flavonoids exhibit antibacterial, antiviral, antioxidant, and antispasmodic properties [50].

### 3.3. Quantitative Evaluation of Phenols Using High-Performance Liquid Chromatography (HPLC)

**Figure 2:**
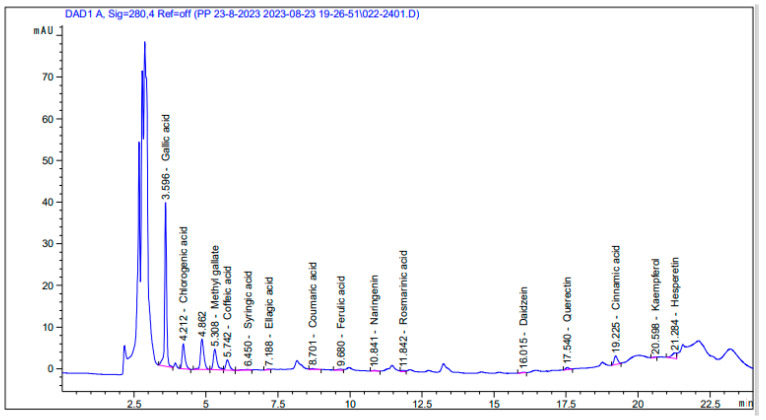
A chromatogram showing the HPLC separation of phenolic compounds in the ethanol extract.

The chromatogram obtained through high-performance chromatography for the ethanol extract of *Chlorella vulgaris* algae reveals a diverse array of compounds (Figure 2). The chromatogram analysis indicates that the extract contains 15 compounds. Identification was performed by comparing their retention durations to the reference phenolic compounds listed in Table 3. Notably, the ethanol extract exhibits substantial quantities of gallic acid and ellagic acid, with respective concentrations of 818.81 and 465.77 µg/g. Conversely, daidzein, kaempferol, and coumaric acid were found to be the least abundant, each registering as 3.34, 3.74, and 5.15 µg/g, respectively. 

### 3.4. Antimicrobial Activity of Chlorella vulgaris Extracts by Well Diffusion Method

The antimicrobial activity for different concentrations of *C. vulgaris* extracts against three pathogenic bacteria, as used in this study, were measured by an agar well diffusion method and the results are presented in Table 4. Paper discs containing 0.01 mg of penicillin were used as standard antibiotics for comparison.

The table presents the results of an antimicrobial activity assay using different extracts against the following three bacterial species: *E. coli*, *Staphylococcus aureus*, and *Bacillus subtilis*. The extracts used were ethanol (95% and 50%) and water, with varying concentrations (2.5 mg/well and 5.0 mg/well). The inhibition zones were measured in millimeters (mm).

Penicillin, used as a positive control, shows significant antimicrobial activity against all three bacterial species, with inhibition zones ranging from 14 ± 0.73 mm to 18 ± 0.90 mm. The 95% ethanol extract shows significant antimicrobial activity against all three bacterial species. The inhibition zones increase with increasing concentrations, indicating a dose-dependent effect. The highest inhibition zones are observed against *E. coli* (12 ± 0.82 mm at 5.0 mg/well), followed by *B. subtilis* (10 ± 0.16 mm at 5.0 mg/well) *and Staphylococcus aureus* (10 ± 1.06 mm at 5.0 mg/well). The 50% ethanol extract also exhibits antimicrobial activity, though slightly lower than that of the 95% ethanol extract. The inhibition zones are highest against *B. subtilis* (13 ± 0.98 mm at 5.0 mg/well), followed by *E. coli* (11 ± 1.80 mm at 5.0 mg/well) and *Staphylococcus aureus* (10 ± 0.49 mm at 5.0 mg/well). The water extract shows minimal antimicrobial activity, with inhibition zones only observed against *Staphylococcus aureus* and *B. subtilis*. The water extract contains the specific components primarily responsible for the antimicrobial effect against Gram-positive bacteria (*S. aureus* and *Bacillus subtilis*), as no effect was observed against Gram-negative bacteria (*E. coli*).The results agree with [51], who stated that the water extract of *C. vulgaris* has no inhibitory zone on *E.coli*.

The inhibition zones are significantly lower compared to the ethanol extracts, indicating that the antimicrobial compounds are more soluble in ethanol than in water. The results are consistent with the findings reported by [52,53].

The results also showed that *C. vulgaris* extracts have a lower effect on bacterial growth than penicillin at a concentration of 0.01 mg/well.

The *Chlorella vulgaris* extract, besides being rich in phenolics and flavonoids, has also been reported to possess antimicrobial properties through many mechanisms that disrupt the structure and functioning of microbial cells, including the disruption of microbial membranes and enzymes, the induction of oxidative stress, metal chelation, the interference with nucleic acid synthesis, and the inhibition of efflux pump activity. Therefore, the combined action of all the mechanisms mentioned may cause the effective inhibition of growth [14,26,54].

### 3.5. Antioxidant Assays

The phenolic compounds’ antioxidant action is attributable to their oxidative–regenerative activity as reference agents or donors of hydrogen and scavengers of O_2_, as well as their ability to chelate the minerals [8]. Several experiments were used to assess the antioxidant activity for different *C. vulgaris* extracts in vitro. Phosphomolybdate, DPPH^•^, and ABTS^•^ testing are examples of these procedures, which are based on staining and depigmentation at a specified wavelength.

#### Phosphomolybdate Assay

Table 5 presents the total antioxidant capacity (TAC) of the various extracts and *Chlorella vulgaris* powder. The TAC is measured in milligrams of ascorbic acid equivalent per gram of extract (mg AAE/g). The table shows that the water extract has the highest TAC with a value of 91.6 mg AAE/g, followed by the 95% ethanol extract with a value of 49.6 mg AAE/g. The 50% ethanol extract has a TAC of 63.2 mg AAE/g, which is significantly lower than the water extract but higher than the 95% ethanol extract. The *C. vulgaris* powder has a TAC of 57.1 mg AAE/g, which falls between the 50% ethanol and 95% ethanol extracts. The results suggest that the water extract has the highest antioxidant capacity, followed by the 95% ethanol extract. The 50% ethanol extract has a lower antioxidant capacity compared to the water extract but is higher than the 95% ethanol extract. The *C. vulgaris* powder has an antioxidant capacity that is intermediate between the 50% ethanol and 95% ethanol extracts.

These findings are consistent with previous studies that have shown that water extracts of *Chlorella vulgaris* have higher antioxidant capacities compared to ethanol extracts. The higher antioxidant capacity of the water extract may be due to the solubility of the antioxidant compounds in water, which allows for better the extraction and preservation of these compounds [55,56].

Compared to the study by [55], which tested the total antioxidant capacity (TAC) by the phosphomolybdate method on *C. vulgaris*, (Table 5) the total oxidative capacity of the aqueous extract was also higher than that of the alcoholic extracts, and this is consistent with what was reported here.

### 3.6. Burger Fortification with Chlorella vulgaris Powder

#### 3.6.1. Chemical Composition of Burger Samples

The determination of the moisture content, protein content, ether extract, and ash content were conducted, and the findings are shown in Table 6. The moisture content for all burger treatments exhibited a range of 64.15% to 64.88%. Notably, the initial moisture content of most burger samples was approximately 60%, indicating compliance with the Egyptian standard specifications.

The protein contents of the burger samples varied from 22.45% to 25.00% at the initial time. These results aligned with the Egyptian standard specifications, which stipulate a minimum protein content of 15% for burgers. The addition of C. vulgaris powder resulted in an increase in protein content by 22.45%, 23.68%, 24%, and 25% for the control, and by 0.5%, 1%, and 1.5% for the additions of *C. vulgaris* powder, respectively. This augmentation can be attributed to the elevated protein content inherent in *C. vulgaris.*

The ether extract in the burger samples ranged from 14.24% to 14.57% at the initial time point, falling below the levels specified by the Egyptian standards.

The ash content of the burger samples varied between 2.72% and 3.33%, all of which were below the Egyptian standard specifications. The highest ash percentage was observed in the samples containing *C. vulgaris*, with an increasing trend corresponding to the addition rate. This rise may be attributed to the heightened mineral content of *C. vulgaris* powder. Interestingly, all treatments exhibited higher ash percentages than the control, indicating the presence of additional minerals in the additives; this result agrees with [57].

The fortification of the burger samples with *C. vulgaris* powder increased the ash and protein contents of the burger samples; the protein and ash contents of the burger samples increased with an increasing *C. vulgaris* powder percentage. Because the *C. vulgaris* powder provides a lot of minerals and protein, adding it to meat products increases the ash content. These results are consistent with [58], who found that adding honey and white chlorella (3%) led to a significant increase in the ash levels and the protein content of frankfurter samples compared to the control.

#### 3.6.2. Microbiological Quality Standards for Burger Samples Stored Frozen for Six Months

Meat products are susceptible to contamination and the proliferation of microorganisms, leading to both spoilage and the potential for the transmission of foodborne diseases.

#### 3.6.3. Change in the Total Bacterial Count of Burger Samples during Storage

The impact of various concentrations (0%, 0.5%, 1%, and 1.5%) of *C. vulgaris* powder on the total bacterial count in the burgers over a storage period of 0, 1, 2, 3, 4, 5, and 6 months at −20 °C is detailed in Table 7. The results presented in Table 7 illustrate the logarithmic influence of *C. vulgaris* powder on the total bacterial count in the burger samples stored at −20 °C for varying durations.

The logarithmic values of total bacteria for the 0.5% *C. vulgaris* treatment were recorded as 5.03, 4.90, 4.70, 4.30, 3.40, 3.00, and 2.90 for the respective storage durations. Correspondingly, the 1% *C. vulgaris* treatments exhibited values of 5.60, 4.70, 4.70, 3.80, 3.20, 2.90, and 2.70. The 1.5% *C. vulgaris* treatments demonstrated logarithmic values of 5.05, 4.80, 4.60, 3.40, 3.00, 2.80, and 2.60. In contrast, the control showed values of 5.10, 5.06, 4.96, 4.90, 4.30, 4.10, and 3.40, respectively.

The findings suggest that *C. vulgaris* powder exerted a concentration-dependent effect, with an observable decrease in logarithmic numbers as the concentration increased. Importantly, the reduction in bacterial numbers was more pronounced compared to the control. Consequently, it can be inferred that the application of *C. vulgaris* powder has the ability to prolong the shelf life of burgers.

#### 3.6.4. Change in Total Yeast and Mold Count of Burger Samples during Storage

Table 7 presents the impact of incorporating *C. vulgaris* powder on the total yeast and mold count of the burger samples in comparison with the control throughout a 6-month storage period at −20 °C. The results, as depicted in Table 7, reveal a notable reduction in the logarithmic yeast and mold count with the addition of *C. vulgaris*, surpassing the control group over the entire storage duration.

Table 7 further elucidates the logarithmic values of the total yeast and mold count for different concentrations of *C. vulgaris* (0.5%, 1%, and 1.5%) and the control at varying time points (0, 1, 2, 3, 4, 5, and 6 months). Specifically, the 0.5% *C. vulgaris* treatments exhibited logarithmic values of 3.81, 3.70, 3.20, 2.80, 2.70, 1.40, and 1.30 for the respective storage periods. Similarly, the 1% *C. vulgaris* treatments demonstrated values of 3.81, 3.60, 3.00, 2.70, 2.50, 1.30, and 1.17. The 1.5% *C. vulgaris* treatments displayed logarithmic values of 3.81, 3.60, 2.90, 2.80, 2.30, 1.20, and 1.11. Notably, an inverse relationship between the concentration and the logarithmic total yeast and mold count was observed during the storage period of the frozen burger samples. This implies that the application of *C. vulgaris* powder has the potential to extend the preservation period of food.

#### 3.6.5. Change in Total Coliform Group Count of Burger Samples during Storage for Six Months

Table 7 provides insights into the impact of incorporating *C. vulgaris* powder on the logarithm of the total Coliform group count in the burger samples, comparing these effects with the control, over a storage period of 0, 1, 2, 3, 4, 5, and 6 months at −20 °C. Table 7 illustrates the logarithmic modulation of the total Coliform group count in the burger samples as influenced by *C. vulgaris* during the entire storage duration.

The logarithmic values of the total coliform group count for the 0.5% *C. vulgaris* treatments were recorded as 4.11, 3.80, 3.70, 3.50, 3.30, 3.20, and 3.10 for the respective storage periods. In parallel, the 1% *C. vulgaris* treatments exhibited values of 4.11, 3.80, 3.50, 3.40, 3.30, 3.00, and 2.80. The 1.5% *C. vulgaris* treatments demonstrated logarithmic values of 4.12, 3.80, 3.50, 3.30, 3.20, 2.70, and 2.50. In comparison, the control showed values of 4.05, 4.00, 3.88, 3.70, 3.70, 3.38, and 3.20, respectively.

It is noteworthy that the reduction in the logarithm of the total Coliform group count was more substantial in the *C. vulgaris* treatments than in the control group after 6 months of storage at −20 °C. This implies that *C. vulgaris* has the potential to be utilized for extending the shelf life of burgers against the Coliform group.

#### 3.6.6. Change in Total Staph Count of Burger Samples during Frozen Storage for 6 Months

The alteration in the total staph count of the burger samples during a 6-month frozen storage period is detailed in Table 7. This table highlights the influence of the concentrations of *C. vulgaris* powder and a control group on the logarithmic total staph count in the burgers over the course of 0, 1, 2, 3, 4, 5, and 6 months of storage at −20 °C

Table 7 collectively demonstrates the logarithmic impact of *C. vulgaris* on the total staph count in the burger samples. Specifically, the logarithmic values of the total staph count for the 0.5% *C. vulgaris* treatments were observed to be 4.47, 4.41, 4.08, 3.90, 2.20, 2.00, and 1.90 over the respective storage periods. Correspondingly, the 1% *C. vulgaris* treatment exhibited values of 4.45, 4.27, 4.08, 3.80, 2.14, 1.90, and 1.70. The 1.5% *C. vulgaris* treatments displayed logarithmic values of 4.46, 4.23, 3.90, 3.60, 2.00, 1.80, and 1.60. In contrast, the control group demonstrated values of 4.50, 4.46, 4.40, 4.20, 3.95, 3.80, and 3.50, respectively.

It is evident from the results that the logarithm of the total staph count decreased with an increase in the *C. vulgaris* powder concentration during the storage period of the frozen burgers. The magnitude of the reduction in the logarithm of the total staph count for the *C. vulgaris* treatments was notably higher than that observed in the control by the end of the storage period. These results indicate the potential of *C. vulgaris* powder to be applied to extend the shelf life of burgers against staph contamination.

### 3.7. Chemical Quality Standards of Burger Samples Fortified with C. vulgaris Powder during Frozen Storage for Six Months

#### 3.7.1. Change in TBA Values of Burger Samples during Storage

**Figure 3:**
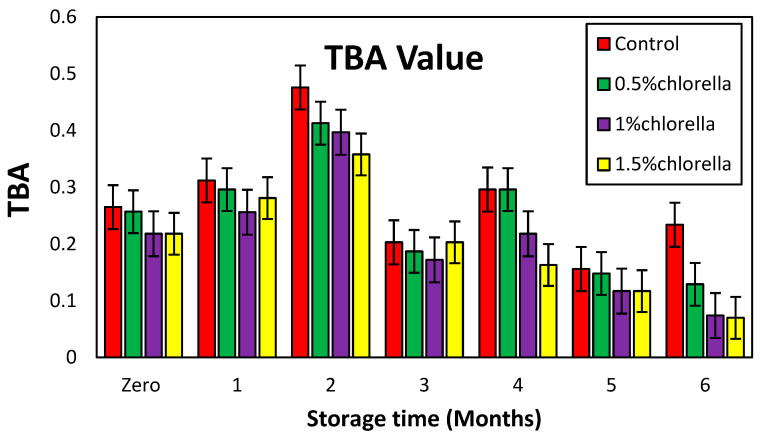
Change in the TBA values of burger samples with *C. vulgaris* powder during storage.

In the investigation of the burger samples subjected to frozen storage at −20 °C for 0, 1, 2, 3, 4, 5, and 6 months, fluctuations in the TBA values (expressed as mg malonaldehyde/kg meat) were observed and are presented in Figure 3. Throughout the storage period, the TBA values for all of the samples exhibited oscillations, suggesting a dynamic interplay possibly arising from the condensation of malonaldehyde with deteriorated compounds derived from proteins, so additional approaches based on tertiary lipid oxidation products are advised [59].

The results indicated that the TBA value is not a suitable method for determining oxidative rancidity in food, despite being an excellent method for measuring oxidative rancidity in fats and oils. This may be because food is a complex medium.

#### 3.7.2. Change in TVBN (Total Volatile Basic Nitrogen) Values of Burger Samples Fortified with *C. vulgaris* Powder during Storage

**Figure 4:**
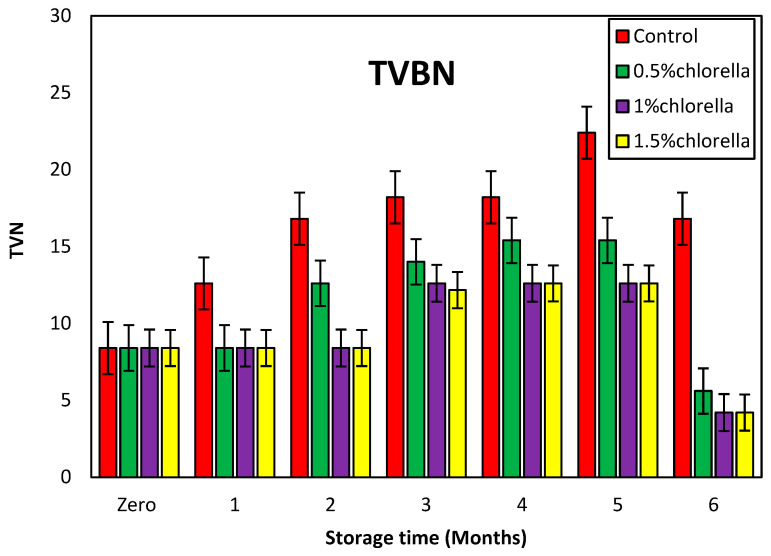
Change in TVBN values of burger samples with *C. vulgaris* during storage.

The variations in the TVBN values, utilized as a metric for assessing protein degradation and employed in this study of burger samples throughout storage at −20 °C for 0, 1, 2, 3, 4, 5, and 6 months, were investigated, and the results are shown in Figure 4.

The findings reveal that the TVBN content of the burger samples incorporating *C. vulgaris* powder and stored at −20 °C exhibited distinct trends over the 0–6-month period. Specifically, the TVBN values for the samples enriched with 0.5% *C. vulgaris* were 8.4, 8.4, 12.6, 14.0, 15.4, 15.4, and 5.6, respectively. Meanwhile, the treatments with 1% and 1.5% *C. vulgaris* displayed the same values of 8.4, 8.4, 8.4, 12.6, 12.6, 12.6, and 4.2. Notably, the TVBN values for these concentrations were consistently lower compared to the control. Additionally, it was observed that both the 1% and 1.5% concentrations exerted a similar effect on the TVBN values.

These results suggest a robust inhibitory effect of *C. vulgaris* powder on proteolytic microorganisms, substantiating its potential as a preservative in mitigating protein degradation during frozen storage.

#### 3.7.3. Change in Acidity Percentages of Burger Samples during Storage

**Figure 5:**
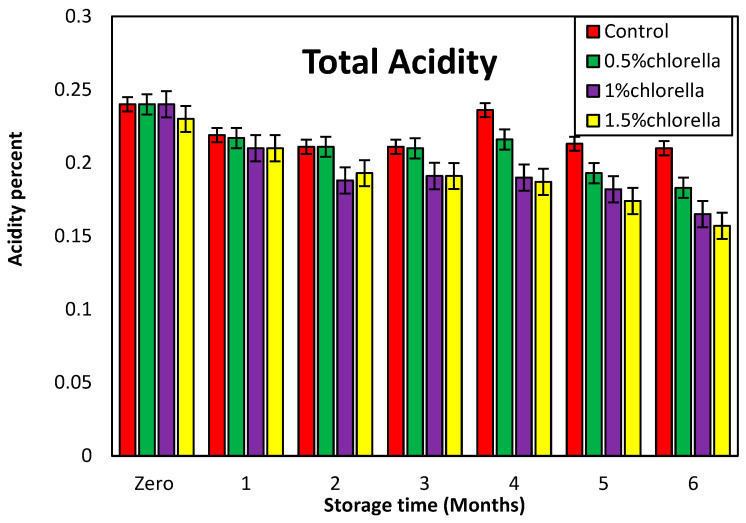
Change in the acidity percentages of burger samples with *C. vulgaris* powder during storage for six months.

Figure 5 presents the total acidity levels of the burger samples subjected to freezing at −20 °C for durations ranging from 0 to 6 months. Notably, the figure reveals a decline in the acidity with prolonged storage, and this decrease is more pronounced in the burgers treated with *C. vulgaris* powder compared to the control, which increased at the end of the storage period.

The recorded results depict a fluctuation in the acidity percentages, with values oscillating moderately between high and low levels throughout the storage period. Despite the slight variations between the control and treated samples during this time frame, a consistent observation is that, at the end of the storage period, the acidity percentages for all treatments are lower than those for the control. This underscores the impact of *C. vulgaris* powder in effectively reducing the acidity, suggesting its potential role in preserving the acidity levels of burger samples during frozen storage.

#### 3.7.4. Change in pH Values of Burger Samples during Storage

**Figure 6:**
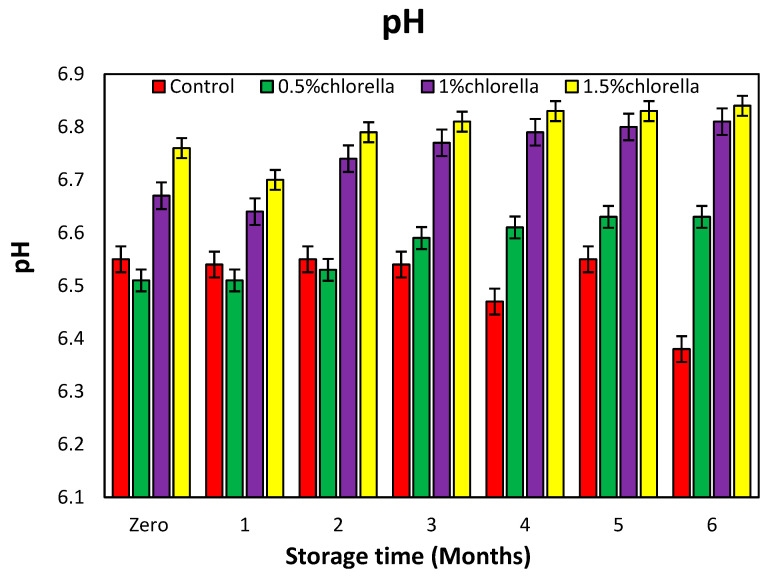
Change in pH values of burger samples with *C. vulgaris* powder during storage.

The alterations in the pH values of the burger samples subjected to storage at −20 °C for 0, 1, 2, 3, 4, 5, and 6 months were systematically investigated, and the outcomes are depicted in Figure 6.

Examination of the data in Figure 6 reveals an upward trend in the pH values with prolonged storage for the treated samples compared to control sample, although a decline is observed in the fifth and sixth months for the control. Particularly noteworthy is the discernible impact of *C. vulgaris* on the pH values, with a clear escalation correlating with increased concentrations, as illustrated in Figure 6.

This observed influence implies that the additives, particularly *C. vulgaris*, engendered a potent antimicrobial effect, resulting in reduced microbial activity. This, in turn, led to an increase in the pH. The relationship between the microbial activity and pH aligns with findings from [60], indicating an inverse correlation between the pH values and the abundance of microorganisms. The current study underscores the role of additives, especially *C. vulgaris*, in modulating pH dynamics and influencing the microbial activity in the stored burger samples.

In addition, the pH of chlorella may be slightly alkaline, depending on its mineral, polyphenol, sugar, and flavonoid contents. Therefore, when applied to a meat product, the pH can be affected. This agrees with [58], who discovered that adding honey and white chlorella to frankfurters raises the pH level.

## 4. Conclusions

In conclusion, our study demonstrates that the incorporation of *Chlorella vulgaris* (C.V) into beef burgers significantly impacts their nutritional composition and quality characteristics. The specific solvent used to extract active substances from C.V plays a role in the concentration of beneficial compounds, with the water extract exhibiting the highest total phenolic and flavonoid contents, and ethyl acetate exhibiting the strongest antimicrobial activity. The addition of C.V at fortification levels of 0.5%, 1%, and 1.5% showed promising results in enhancing the nutritional quality of beef burgers. Specifically, the treatments with C.V exhibited improved microbial stability, with lower microbial contents compared to the control, and better control over TVN values, indicating reduced lipid oxidation. The pH values were slightly higher in the treatments, which is desirable to inhibit pathogenic bacterial growth, and the total acidity was also reduced. Therefore, it can be concluded that the incorporation of C.V into beef burgers at a concentration of 0.5% provides a viable strategy to enhance the nutritional profile of the product.

Finally, based on the results obtained, adding chlorella powder at concentrations of 0.5% and 1% to the beef burger reduced the microbial content as a result of it containing phenolic and flavonoid substances that have an antibacterial and antioxidant effects. The burger contained more protein and some nutrients, which increased the nutritional value and maintained its quality for a longer period. In conclusion, utilizing *Chlorella vulgaris* algae as a natural preservative to extend the freshness of burgers is a sustainable and innovative approach to food preservation. By harnessing the power of this green superfood, we not only enhance the shelf life of our food products but also contribute to a healthier and more environmentally friendly food industry. Further research could build upon these findings by investigating the specific mechanisms through which C.V improves the quality characteristics of beef burgers and exploring the potential synergistic effects with other natural fortificants.

## Figures and Tables

**Table 1:** Chemical composition of *Chlorella vulgaris* (% dry weight).

Parameter	Values
Moisture	10.00 ± 0.17
Ash	13.00 ± 0.24
Crude protein	38.17 ± 0.08
Carbohydrate	21.40 ± 0.17
Crude ether extract	8.08 ± 0.07
Crude fiber	9.35 ± 0.06
P (mg/g)	79.80 ± 1.14
Ca (mg/g)	0.19 ± 0.01
Na (mg/g)	51.30 ± 0.90
K (mg/g)	8.00 ± 0.16
Mg (mg/g)	2.70 ± 0.33
Fe (µg/g)	1220 ± 28.58
Mn (µg/g)	46.00 ± 3.27
Zn (µg/g)	368 ± 5.72
Cu (µg/g)	20.00 ± 0.82
Total Chlorophyll (mg/g)	38.16 ± 0.22
Carotenoids	17.56 ± 0.24

Values are means ± standard deviation.

**Table 2:** Total phenols (mg gallic acid/g extract) and total flavonoids (mg quercetin/g extract) in the *Chlorella vulgaris* extracts.

Extracts	Yield Extract (%)	Total Phenols (mg GAE/g)	Total Flavonoids (mg QE/g)
Ethanol 95%	25.7 ± 0.49	127.7 ± 0.90	37.4 ± 1.22
Water	33.3 ± 0.65	183.5 ± 0.81	54.0 ± 0.82
Ethanol 50%	29.0 ± 0.57	145.5 ± 2.16	51.3 ± 0.41

Values are means ± standard deviation.

**Table 3:** The most important phenolic compounds separated by HPLC from the ethanol extract.

Components	Area	Conc. (µg/g)
Gallic acid	182.62	818.81
Chlorogenic acid	41.99	290.23
Methyl gallate	41.97	110.90
Caffeic acid	21.11	88.22
Syringic acid	2.69	10.80
Ellagic acid	1.39	465.77
Coumaric acid	2.72	5.15
Ferulic acid	2.74	21.42
Naringenin	1.23	46.64
Rosmarinic acid	3.25	18.30
Daidzein	1.13	3.34
Quercetin	4.02	73.98
Cinnamic acid	17.70	16.61
Kaempferol	1.19	3.74
Hesperidin	14.08	31.98

**Table 4:** Antibacterial activity of *Chlorella vulgaris* extracts.

Extracts	Conc. (mg/Well)	Inhibition Zone (mm)		
		*E. coli*	*Staphylococcus aureus*	*Bacillus subtilis*
Ethanol 95%	2.5	9 ± 1.63	6 ± 0.98	8 ± 1.14
	5.0	12 ± 0.82	10 ± 1.06	10 ± 0.16
Water	2.5	-	4 ± 0.33	2 ± 0.20
	5.0	-	10 ± 1.80	3 ± 0.29
Ethanol 50%	2.5	8 ± 0.98	6 ± 0.41	11 ± 0.18
	5.0	11 ± 1.80	10 ± 0.49	13 ± 0.98
Penicillin	0.01	18 ± 0.90	15 ± 0.82	14 ± 0.73

Values are means ± standard deviation.

**Table 5:** Total antioxidant capacity of *Chlorella vulgaris* extracts and powder (mg AAE/g Ex).

Type of Extract Samples	TAC (mg AAE/g)
Ethanol 95% extract	49.6 ^d^ ± 1.06
Water extract	91.6 ^a^ ± 1.14
Ethanol 50% extract	63.2 ^b^ ± 0.24
*C. vulgaris* powder	57.1 ^bc^ ± 0.65

Values are means ± standard deviation; the means within each column designated with the same letter are not significant differences (*p* > 0.05).

**Table 6:** Proximate chemical composition of burger samples.

Treatments	Composition%			
	Moisture	Protein	Ether Extract	Ash
Control	64.49 ± 0.20	22.45 ± 0.06	14.24 ± 0.24	2.72 ± 0.25
0.5% Algae	65.15 ± 0.05	23.68 ± 0.48	14.36 ± 0.13	2.92 ± 0.07
1% Algae	64.50 ± 0.08	24.00 ± 0.06	14.29 ± 0.25	3.14 ± 0.04
1.5% Algae	64.88 ± 0.54	25.00 ± 0.19	14.57 ± 0.34	3.33 ± 0.04
ESS	≤60	≥15	≤20	≤5

ESS = Egyptian Standard Specification; values are means ± standard deviation.

**Table 7:** Change in microbial counts (log_10_ cfu/g) of burger samples during frozen storage for 6 months.

Parameters	Storage Time (Months)	Treatments			
		Control	0.5%C.V	1%C.V	1.5%C.V
Total bacterial count (log_10_ cfu/g)	0	5.10 ± 0.16	5.03 ± 0.30	5.06 ± 0.10	5.05 ± 0.08
	1	5.06 ± 0.46	4.90 ± 0.24	4.70 ± 0.34	4.80 ± 0.42
	2	4.96 ± 0.78	4.70 ± 0.57	4.70 ± 0.57	4.60 ± 0.18
	3	4.90 ± 0.33	4.30 ± 0.16	3.80 ± 0.12	3.40 ± 0.22
	4	4.30 ± 0.57	3.40 ± 0.08	3.20 ± 0.18	3.00 ± 0.08
	5	4.10 ± 0.08	3.00 ± 0.73	2.90 ± 0.13	2.80 ± 0.24
	6	3.40 ± 0.33	2.90 ± 0.37	2.70 ± 0.42	2.60 ± 0.20
	Reduction%	33.33	42.35	46.64	48.51
Total yeast and mold count (log_10_ cfu/g)	0	3.82 ± 0.26	3.81 ± 0.29	3.81 ± 0.26	3.81 ± 0.07
	1	3.80 ± 0.05	3.70 ± 0.19	3.60 ± 0.29	3.60 ± 0.12
	2	3.30 ± 0.43	3.20 ± 0.19	3.00 ± 0.10	2.90 ± 0.11
	3	3.10 ± 0.12	2.80 ± 0.22	2.70 ± 0.12	2.80 ± 0.03
	4	3.10 ± 0.22	2.80 ± 0.16	2.50 ± 0.02	2.30 ± 0.07
	5	3.00 ± 0.14	1.40 ± 0.07	1.30 ± 0.11	1.20 ± 0.06
	6	2.90 ± 0.16	1.30 ± 0.01	1.17 ± 0.07	1.11 ± 0.06
	Reduction%	24.08	65.88	69.29	70.87
Total Coliform group count (log_10_ cfu/g)	0	4.00 ± 0.22	4.11 ± 0.04	4.11 ± 0.07	4.12 ± 0.07
	1	4.00 ± 0.29	3.80 ± 0.42	3.80 ± 0.10	3.80 ± 0.03
	2	3.88 ± 0.33	3.70 ± 0.07	3.50 ± 0.18	3.50 ± 0.09
	3	3.70 ± 0.34	3.50 ± 0.20	3.40 ± 0.25	3.30 ± 0.11
	4	3.70 ± 0.16	3.30 ± 0.18	3.30 ± 0.10	3.22 ± 0.12
	5	3.38 ± 0.21	3.20 ± 0.19	3.00 ± 0.10	2.70 ± 0.07
	6	3.20 ± 0.25	3.10 ± 0.20	2.80 ± 0.12	2.50 ± 0.21
	Reduction%	20.99	24.57	31.87	39.32
Total *Staphylococcus* count (log_10_ cfu/g)	0	4.50 ± 0.12	4.47 ± 0.16	4.45 ± 0.35	4.46 ± 0.15
	1	4.46 ± 0.17	4.41 ± 0.22	4.27 ± 0.19	4.23 ± 0.23
	2	4.40 ± 0.23	4.08 ± 0.08	4.08 ± 0.13	3.90 ± 0.15
	3	4.22 ± 0.20	3.90 ± 0.33	3.80 ± 0.15	3.60 ± 0.09
	4	3.95 ± 0.13	2.20 ± 0.19	2.14 ± 0.11	2.00 ± 0.17
	5	3.80 ± 0.18	2.00 ± 0.14	1.90 ± 0.11	1.80 ± 0.02
	6	3.50 ± 0.11	1.90 ± 0.07	1.70 ± 0.15	1.60 ± 0.07
	Reduction%	22.22	57.47	61.79	64.13

C.V = *Chlorella vulgaris*; Reduction% = ((log total count in zero time—log total count in end time)/log total count in zero time)) × 100; values are means ± standard deviation.

## Data Availability

The original contributions presented in the study are included in the article, further inquiries can be directed to the corresponding author.
